# Developing a competency framework for training with simulations in healthcare: a qualitative study

**DOI:** 10.1186/s12909-024-05139-1

**Published:** 2024-02-23

**Authors:** Uroš Zafošnik, Venija Cerovečki, Nataša Stojnić, Anja Poženel Belec, Zalika Klemenc-Ketiš

**Affiliations:** 1 Ljubljana Community Health Centre, Metelkova 9, 1000 Ljubljana, Slovenia; 2 Department of Family Medicine, School of Medicine, University of Zagreb, 10000 Zagreb, Croatia; 3 Health Centre Zagreb-Centar, 10000 Zagreb, Croatia; 4 Department of Family Medicine, Medical Faculty, University of Maribor, Taborska 8, 2000 Maribor, Slovenia; 5 Department of Family Medicine, Medical Faculty, University of Ljubljana, Poljanski nasip 58, 1000 Ljubljana, Slovenia

**Keywords:** Method, Teaching, Competence, Delphi study, Simulation, Healthcare

## Abstract

**Background:**

Simulation is a technique used to create an experience without going through the real event. Competency-based medical education focuses on outcomes and ensures professionals have the necessary knowledge, skills, and attitudes. The purpose of this study was to develop a set of competencies for the instructors providing basic and advanced levels of simulation-based training in healthcare.

**Methods:**

We conducted a qualitative study in three steps, with each next step building on and influenced by the previous one. First, we conducted a literature review, then a consensus development panel, and finally a three-step Delphi process. The participants were experts in the fields of healthcare, education, and simulations.

**Results:**

The six main competencies identified for the instructor providing simulation-based training at the basic level in healthcare include knowledge of simulation training, education/training development, education/training performance, human factors, ethics in simulation, and assessment. An instructor providing simulation-based training at an advanced level in healthcare should also possess the following five competencies: policies and procedures, organisation and coordination, research, quality improvement, and crisis management.

**Conclusion:**

The identified competencies can serve as a valuable resource for simulation educators and organisations involved in simulation education, to plan curriculum and implement a continuous train-the-trainers programme.

**Supplementary Information:**

The online version contains supplementary material available at 10.1186/s12909-024-05139-1.

## Introduction

Simulation is a method or technique used to create an experience without going through the real event [1]. Competency-based simulation training is becoming an increasingly popular method for health professionals to develop and refine their clinical skills in a safe and controlled environment. This approach uses highly authentic simulators and scenarios that closely replicate real-world clinical situations, allowing learners to practice and master specific competencies before applying them in a clinical setting [2]. According to WHO [3], health professional education and training institutions should use simulation methods with a contextual level of realism/fidelity in the training of health professionals, using high fidelity methods in institutions with adequate resources and lower fidelity methods in resource-limited institutions.

Competency is the observable ability of a professional and includes various components such as knowledge, skills, values, and attitudes [4, 5]. Competency-based medical education (CBME) is necessary because it focuses on the outcomes of medical education and training, not just the inputs or related activities. CBME aims to ensure that medical professionals have the knowledge, skills, and attitudes necessary to provide high-quality patient care throughout their careers [6].

Competency models are an effective tool for developing employee performance. Their use is particularly important in personnel selection, planning of evaluation and training processes. The main benefits of developing and using competency models are improved performance, higher quality in developing training programs, optimization of evaluation systems, reasonable recruitment of personnel, and more reliable prediction of employee performance [7].

Effective simulation-based teaching requires a specific set of competencies beyond those required for traditional classroom teaching. These include the ability to design and create effective simulation scenarios, facilitate debriefing and feedback, assess learner competencies, and integrate simulation-based teaching into the general curriculum [8]. Simulations are increasingly used in healthcare education to teach cognitive, psychomotor, and affective skills in individuals and teams.

Simulation-based training in healthcare has some limitations. These include the cost of such training due to expensive equipment, difficulty in achieving realism, technical issues, lack of knowledge about using simulation as a teaching method, standardisation, limited variety of cases used for simulation, limited assessment of soft skills, and time constraints [9, 10]. To minimise the impact of these limitations in practise, professionally trained instructors with the required competencies are needed.

When we talk about the training of trainers delivering training, we are talking about two levels of such training.

In any training, there are two levels of it, basic and advanced. One level is the training of trainers who teach learners in general, and the second level is the training of trainers who teach a group of people who then take on the role of trainers. Such approach is commonly used to ensure the consistent and effective delivery of healthcare training programs [11]. In simulation-based training, the basic level is training instructors to deliver simulation-based teaching to individuals or teams, the advanced level is training instructors to educate other instructors to deliver simulation-based teaching. Both types of instructors need to develop the same basic competencies, which include foundational skills for effective training of healthcare professionals. The instructors providing advanced levels must acquire additional competencies that are crucial for addressing challenges and demands at an advanced level of teaching and leading simulation, including the management of a simulation center [12].

There is a large body of literature describing various training programs that incorporate simulation and their effectiveness [13]. There are also some articles describing frameworks for developing and evaluating such programs [13]. However, to date, we have not been able to find any articles describing the competencies of instructors providing basic and advanced training. Therefore, the purpose of this study was to develop a set of competencies for the instructors providing basic and advanced levels of simulation-based training in healthcare.

## Methods

**Fig. 1:**
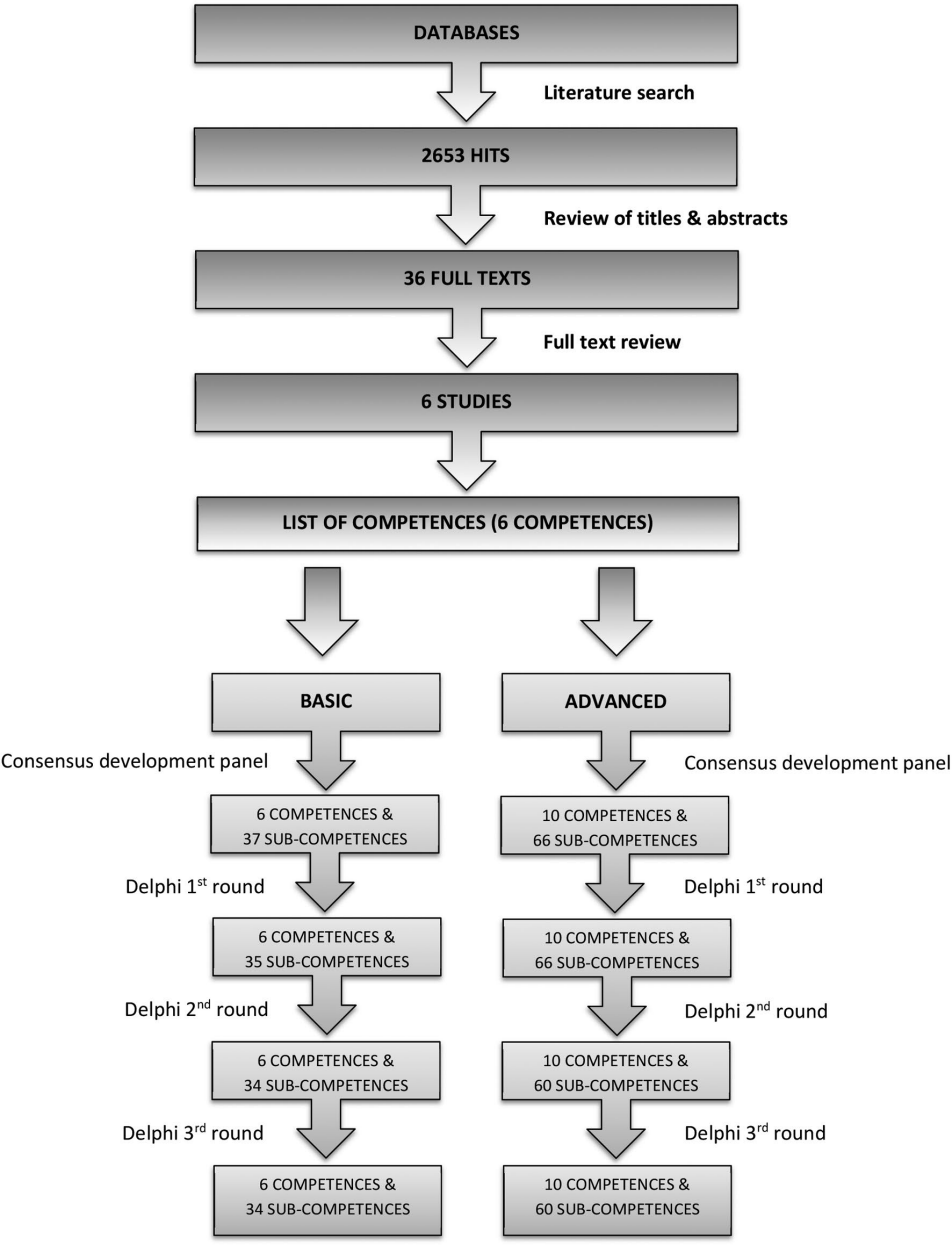
Flow-chart of the study methodology

We conducted a qualitative study in three steps, with each next step building on and influenced by the previous one. First, we conducted a literature review, then a consensus development panel, and finally a three-step Delphi process (Fig. 1). The study took place from the beginning of November 2022 to the end of February 2023.

### Literature review

To determine whether previous studies have already identified the competencies of teachers/instructors using a simulation-based educational approach, we searched four databases: PubMed, Cochrane Library, EMBASE, CINAHL, and Google Scholar. The search was conducted in November 2022 and limited to English-language publications. No restriction on publication date was applied.

We used the following keywords: competency; simulation-based medical education; simulation-based medical learning; simulation-based medical teaching; healthcare; patient simulation; faculty. We chose not to search separately for the competencies of the instructors providing basic and advanced training due to the scarcity of the current literature.

After the initial search, two of the authors (UZ and ZKK) independently reviewed the titles and abstracts, removed duplicates, and excluded the studies that did not qualify for our objective. Next, both researchers independently reviewed the full text of the studies to identify potential competencies. The aim of this process was not to assess the quality of the studies, but only to identify (1) if the competency framework for the instructors providing basic and advanced levels of simulation-based training in healthcare already exist in the literature, and (2) which competencies have already been identified by other authors.

A preliminary list of competencies was generated. Any disagreements between the researchers were resolved through discussion (Fig. 1).

### Consensus development panel

Consensus development panels are a qualitative method for reaching agreement in areas where there is uncertainty or lack of definitive information [14].

We held one round of a consensus development panel. It took place during the consortium meeting of the Transsimed project, held in Ljubljana, Slovenia, November 9–11, 2022. We used a nominal group technique (NGT) that typically includes four main phases: silent generation, roundtable discussion, clarification, and voting/ranking [15, 16]. Following McMillan et al. [16], we replaced the first two phases with a literature review.

The selection of the panel members was purposive to include the experts from all fields (education, simulation, healthcare). There were 10 experts from medical education (3 persons), education with simulation in healthcare (3 persons), and healthcare professionals (physicians, nurses) (8 persons). Some participants were experts in several fields.

The panel members were approached by email. We began the process with a clarification phase where the project leader introduced the scope of the project and relevant definitions, such as competency, training with simulation, methods used in simulation training, and the list of competencies that had emerged from the literature review. Then, participants were asked to review the list of competencies individually and mark each competency according to its relevance, propose possible changes, or propose new competencies. After that, the participants came together in three groups where they clarified, elaborated, defended, or disputed the list of competencies. The process of work was as follows: opening out, exploring, coming together, and reaching a consensus. Afterwards, they presented their ideas to all participants and created a single list of competencies.

The next day, we conducted the fourth phase of the NGT, voting/ranking. Participants were asked to give their opinion on whether a single competency should be included in the final list. We included only those competencies in the final list that received 100% agreement. The voting/ranking phase was followed by a discussion phase where the participants shared opinions on which competence a basic instructor should have and which an advanced instructor should have.

### Delphi process

The Delphi technique is defined as the procedure of asking a panel of experts for their opinion on a relevant issue, summarizing and presenting their collective responses and repeating this process for a certain number of rounds [17]. Using such a process, we aimed to reach consensus among the experts on the final list of competencies for training with simulation.

There were 20 participants purposively invited and all agreed to participate (Table 1). They were approached by email.

**Table 1:** The characteristics of the Delphi process participants*

Characteristic	Physician (N = 13)	Graduated nurse (N = 7)	Academic teacher (N = 11)	Simulation-based education expert (N = 9)
Gender Male Female	5 8	5 2	7 4	5 4
Age category 30–39 years old 40–49 years old 50–59 years old	5 5 3	3 3 1	1 8 2	3 3 3

*Some participants were experts in several fields (for example, a physician was also an academic teacher)

We conducted three rounds of a Delphi procedure using an online approach, with instructions for participation and a link to the electronic survey sent via email. In addition, we sent two reminders for each round.

The first Delphi survey required a simple “yes/no/with changes” response to the inclusion of each competency area, separately for the basic and advanced levels. Participants were then asked to respond “yes/no/with changes” to the inclusion and grouping of each competency. If they indicated that changes were needed, they could write their suggestion in the free text box provided. Items with less than 90% agreement were revised to better align with participant observations.

The second Delphi survey was used to confirm the changes made in the first round. Participants were asked to rate how strongly they agreed with the inclusion of the competency in the model on a scale of one to nine (1 - strongly disagree; 9 - strongly agree). They also had the option to write their comments in the blank space provided. We calculated the mean of their responses, and in the case of < 7 points, the competency was excluded from the list.

The third Delphi survey aimed to reach consensus on the final list of competencies. Participants were again asked to rate on a scale of one to nine (1 - strongly disagree; 9 - strongly agree) how strongly they agreed that the competency should be included in the model. We calculated the mean of their responses and in the case of < 7 points, the competency was excluded from the list.

## Results

### Literature review

After the initial search, we found 2653 abstracts. Of these, 2617 were removed due to overlap and not qualifying for our objective. The full text of the remaining 36 articles was read and 30 were excluded for lack of relevance to our study. After this process, six articles were identified as suitable (Fig. 1). Based on these articles, an initial list of competencies was generated (Table 2), which served as the basis for the consensus development panel.

**Table 2:** The list of main competence categories for the instructors providing basic and advanced levels of simulation-based training in healthcare

Main competence	Literature review	Consensus development panel	Delphi process
Basic level	1. Planning/designing simulations 2. Facilitating learning in safe environments 3. Knowledge based on credible clinical realism 4. Evidence-based knowledge 5. Professional values and identity.	1. Knowledge of simulation training 2. Education/training development 3. Education/training performance 4. Human factors 5. Ethics in simulation 6. Assessment	1. Knowledge of simulation training 2. Education/training development 3. Education/training performance 4. Human factors 5. Ethics in simulation 6. Assessment
Advanced level		1. Knowledge of simulation training 2. Education/training development 3. Education/training performance 4. Human factors 5. Ethics in simulation 6. Assessment 7. Policies and procedures 8. Organisation and coordination 9. Research 10. Quality improvement 11. Crisis management	1. Knowledge of simulation training 2. Education/training development 3. Education/training performance 4. Human factors 5. Ethics in simulation 6. Assessment 7. Policies and procedures 8. Organisation and coordination 9. Research 10. Quality improvement 11. Crisis management

### Consensus development panel

After the consensus development panel, the list of competencies was compiled, one for the basic level and one for the advanced level. The list for the basic level included six main competencies with 37 sub-competencies. The advanced level list included 11 main competencies with 66 sub-competencies (Table 1; Fig. 1).

### Delphi process

At least 65% of the invited experts participated in the Delphi process. In the first round, 20 participated for the basic level and 16 for the advanced level. In the second round, 14/13 participated for the basic/advanced level. In the third round, 16/16 participated for the basic/advanced level.

After the first round of Delphi, we excluded 2 sub-competencies from the basic level model. We also reformulated 16 basic level sub-competencies and 33 advanced level sub-competencies. As a result of the first round of Delphi, the list of basic level competencies included six major competencies with 35 sub-competencies. The list of advanced level competencies included 11 main competencies with 66 sub-competencies (Table 1; Fig. 1).

After the second round of the Delphi process, we deleted one competency for the basic level and reformulated five sub-competencies. For the advanced level, we excluded six sub-competencies and reformulated four of them. This resulted in a list of six main competencies and 34 sub-competencies. This resulted in a list of 11 main competencies and 60 sub-competencies (Table 1; Fig. 1).

**Fig. 2:**
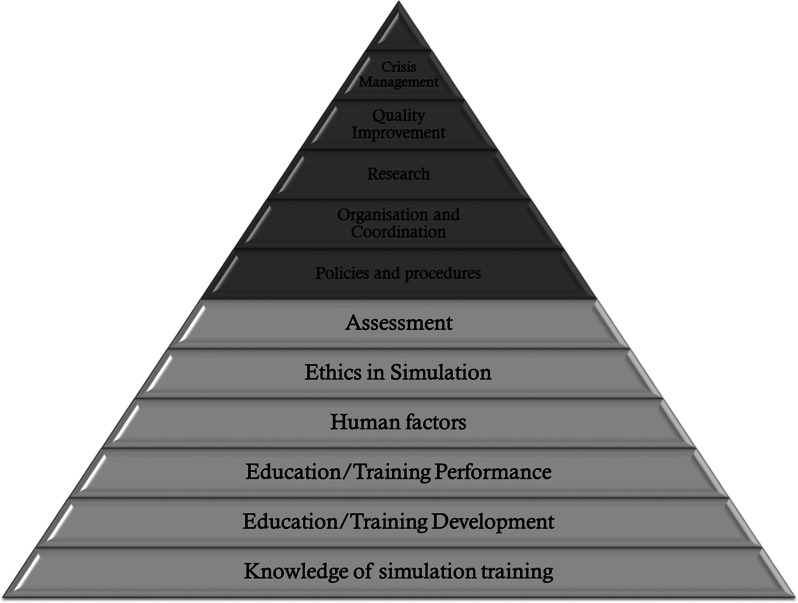
The competencies of instructors providing basic and advanced level simulation-based training within healthcare

In the third round of Delphi, we achieved 100% agreement on all competencies and sub-competencies for both the basic and advanced models. Therefore, the final competency model for the basic level included six main competencies with 35 sub-competencies. The list of competencies at the advanced level included 11 main competencies with 61 sub-competencies (Figs. 1 and 2, Appendix 1).

## Discussion

The six main competencies identified for the instructor providing simulation-based training at the basic level in healthcare include knowledge of simulation training, education/training development, education/training performance, human factors, ethics in simulation, and assessment. An instructor providing simulation-based training at an advanced level in healthcare should additionally possess the following competencies: policies and procedures, organisation and coordination, research, quality improvement, and crisis management.

Healthcare is best learned from experiences in real clinical environment, through interacting with real patients in real clinical situations [18]. However, there are several ethical considerations in such training [19]. When real patients are used for training, it can be difficult to obtain informed consent, especially for invasive or risky procedures. There are issues with patient privacy and data confidentiality.

Simulation-based healthcare education is also a form of experiential learning [20]. Here, knowledge is created through the transformation of experience. Simulation enables students to think critically from experience [21].

Medical professionals can hone their skills through repeated practice on simulators, which can lead to better outcomes for patients when faced with real medical situations [22, 23]. Medical simulation prevents harm to real patients who might otherwise be exposed to unnecessary procedures, misdiagnosis or inexperienced healthcare workers during training [19].

Simulation-based training provides safe learning through creating a psychologically safe learning environment [24, 25].

Simulation-based medical education is a structured process, including the following steps: presentation of theoretical knowledge, skills training, briefing, simulation, and debriefing [26]. The latter is the most important component which enables a systematic reflection on the experience during the simulation [27]. There are many ways in which simulation-based training can be applied, such as low-fidelity simulations, high-fidelity simulations, standardised patients, virtual standardised patients, augmented and virtual reality, serious gaming, and in situ simulations [28].

Therefore, instructors that perform simulation-based training must have a comprehensive range of skills. Previous studies have identified a lack of a conceptual framework for simulation in healthcare training [29]. Our literature review found that there was no comprehensive competency model, especially for the different levels of instructors [8, 30–32]. It is worth mentioning that, due to the lack of articles in this area, we did not perform the search separately for basic and advanced competencies for instructors. We made the separation after the literature review.

The results of this study allowed us to recognise the multidimensional nature of simulation education. The competencies identified cover a wide range of areas, including knowledge of simulation training, education/training development and performance, human factors, ethics in simulation, evaluation, policies and procedures, organisation and coordination, research, and quality improvement. This underscores the need for instructors to have a broad and diverse skill set to be effective in their role.

Our findings indicate that the competence “Knowledge of simulation training” is important for the quality of education. This is consistent with a study that found a positive correlation between healthcare tutors’ knowledge and their engagement in simulation practice, suggesting that as healthcare tutors’ understanding of simulation increases, so does their involvement in simulation activities [33]. Instructors are also expected to master the theory of education in order to be able to develop trainings. We also identified in our study that the competence “Education/Training Development” is needed for instructors providing basic and advanced training. Similarly, an instructor should be able to give lessons, but in the context of simulation-based education, and know different types and methods of simulation training. This was highlighted in our study as the competence “Education/Training Performance” was identified.

Human factors pertain to the diverse elements influencing our individual job performance and our interactions within teams and technology. Human factors improve patient safety [34], and it has been shown that simulation-based training is a successful learning tool to improve qualified healthcare teams’ human factor skills [2]. In line with that, the competence “Human Factors” was identified in our study. The competence “Ethics in simulation” is also a part of the competence framework identified in our study. Ethical considerations permeate almost every element of simulation and clinical practice [35]. Also, an ethical code has been developed for simulation-based training in healthcare [36].

Simulation-based training is already widely used for both formative and summative assessment [37, 38] so our framework contains also the competence “Assessment”.

The competencies discussed above should be mastered by instructors providing basic and advanced training. The competencies that are discussed next, should be mastered by instructors providing advanced training.

Our findings indicate that an advanced instructor should possess the ability to develop policies and procedures specifically tailored for a simulation center, demonstrating competence in “Policies and Procedures.” Namely, the studies show that the use of healthcare simulation can pose safety risks, particularly when implemented “in situ” within actual clinical settings, where the distinction between simulated and real practice may become unclear [39]. A procedure for developing procedures and policies to address such issues has already been developed [39].

The establishment and operation of a simulation center is a comprehensive task and includes not only the development and delivery of training, but also the provision of personnel, maintenance of equipment, procurement and planning of costs, etc. [40]. Therefore, an advanced trainer should have a competence in organization and coordination, identified in our study as “Organisation and Coordination”.

The results of our study showed that the competence “Research” is a part of the framework. The literature shows that research in the field of simulation-based education is essential for the continuous improvement of the quality, effectiveness, and impact of healthcare training practices [41, 42]. It serves as a basis for evidence-based decision making and contributes to the continuous development of healthcare professionals. While there is an extensive literature on simulation, more research is needed to find answers to unexplored questions and to uncover further benefits of simulation as a highly effective teaching and learning approach and to facilitate its full integration into healthcare curricula [23].

Simulations in healthcare can be used to improve quality [43]. For example, a quality department in a healthcare facility identifies several safety incidents. These are then used as the basis for developing simulation-based training to overcome safety issues. Similarly, simulations can be used to test clinical systems using a simulation-based clinical system testing (SbCST) method. This method has the potential to significantly influence the construction and evaluation of healthcare facilities in the future [44]. In line with the above, the competence “Quality Improvement” was identified in our study.

The last competence of the instructors providing advanced training identified in our study was “Crisis Management”. Crisis management refers to a set of principles that address cognitive and interpersonal behaviors that contribute to optimal team performance. Crisis management training using simulation enhances the cognitive and interpersonal skills of medical personnel in simulated environments [45]. In addition, there is growing evidence of the effectiveness of crisis management training programs in improving team performance and mitigating negative patient outcomes in real clinical situations [46].

It is important to note that the identified competencies are not exhaustive and that additional competencies may be required depending on the specific context and type of simulation-based training. For example, simulation-based education can be used in a variety of settings, such as primary care, hospital care, social work, palliative care, etc. [25, 47].

The competencies identified in this study can serve as a valuable resource for simulation educators and for organizations and institutions involved in simulation education. They can also serve as a useful framework for healthcare educators and instructors in healthcare to develop their simulation-based education programs [31, 32]. By understanding the competencies required for effective simulation education, educators can better prepare for their roles, and organizations can develop more effective education programs aligned with these competencies. In addition, this study highlights the importance of continuing professional development for simulation educators to ensure that they are equipped with the knowledge and skills necessary to provide high-quality education and training in this rapidly evolving field.

The strengths of this study include a thorough and systematic approach to identifying and defining competencies for simulation educators. The literature review, consensus development panel, and Delphi process allowed for a comprehensive and evidence-based approach to this task, resulting in a highly refined and agreed-upon list of competencies. Given that 100% agreement was achieved on all competencies and sub-competencies in the third round, the resulting competency model is likely very robust and reflects the consensus opinion of experts in the field.

A possible limitation of this study is the relatively small number of articles identified in the initial literature review. Furthermore, we have not conducted a systematic literature search with a quality assessment of the articles. We are aware there was no significant reduction in the main competency categories after the consensus development panel. This could indicate that the discussion among the panel members was weak, but on the other hand, it could also indicate that the original list of competencies based on the literature review was comprehensive. Another limitation is the lack of testing and evaluation of the model in practice. According to the studies, this is a common methodological flaw in many articles [48].

## Conclusion

Simulation-based training is a method of experiential learning in healthcare and other fields (such as social work) that provides a team approach and a safe learning environment for learners. Instructors must have comprehensive competencies to effectively deliver simulation-based training. This study identified six key competencies for instructors providing simulation-based basic healthcare education and five additional competencies for instructors providing higher level education. The identified competencies can serve as a valuable resource for simulation educators and organisations involved in simulation education.

Further research is needed to validate and refine these competencies in different healthcare settings and contexts.

### Electronic supplementary material

Below is the link to the electronic supplementary material.

The final model for basic and advanced level competencies and sub-competencies for the instructors providing basic and advanced levels of simulation-based training in healthcare

## Data Availability

The datasets used and/or analysed during the current study available from the corresponding author on reasonable request.

## References

[1] So HY, Chen PP, Wong GKC, Chan TTN (2019). Simulation in medical education. J Royal Coll Physicians Edinb.

[2] Abildgren L, Lebahn-Hadidi M, Mogensen CB, Toft P, Nielsen AB, Frandsen TF, Steffensen SV, Hounsgaard L (2022). The effectiveness of improving healthcare teams’ human factor skills using simulation-based training: a systematic review. Adv Simul (London England).

[3] Martins JCA, Baptista RCN, Coutinho VRD, Fernandes MID, Fernandes AM (2018). Simulations in nursing and midwifery education.

[4] Epstein RM, Hundert EM (2002). Defining and assessing professional competence. JAMA.

[5] Ellaway R (2016). CanMEDS is a theory. Adv Health Sci Educ Theory Pract.

[6] Ten Cate O (2013). Nuts and bolts of entrustable professional activities. J Grad Med Educ.

[7] Skorková Z (2016). Competency models in Public Sector. Procedia - Social Behav Sci.

[8] Topping A, Bøje RB, Rekola L, Hartvigsen T, Prescott S, Bland A, Hope A, Haho P, Hannula L (2015). Towards identifying nurse educator competencies required for simulation-based learning: a systemised rapid review and synthesis. Nurse Educ Today.

[9] Sørensen JL, Østergaard D, LeBlanc V, Ottesen B, Konge L, Dieckmann P, Van der Vleuten C (2017). Design of simulation-based medical education and advantages and disadvantages of in situ simulation versus off-site simulation. BMC Med Educ.

[10] Henriksen K, Rodrick D, Grace EN, Brady PJ. Challenges in health care simulation: are we learning anything new? Acad Med 2018, 93(5). 10.1097/ACM.0000000000001891 28817431

[11] Buchanan J, Maagaard R, Sammut MR, Windak A (2016). EURACT– a sustainable model for the development of teachers of General Practice/Family Medicine [GP/FM]. Educ Prim Care.

[12] Herrington A, Gupta V. Roles and responsibilities of a medical simulation center manager. In: *StatPearls* StatPearls Publishing; 2023. 32491602

[13] O’Brien B, Bevan K, Brockington C, Murphy J, Gilbert R (2023). Effects of simulation-based cardiopulmonary and respiratory case training experiences on interprofessional teamwork: a systematic review. Can J Respiratory Therapy: CJRT = Revue canadienne de la Ther respiratoire: RCTR.

[14] Green J, Thorogood N. Qualitative methods for health research. In. London: SAGE Publications Ltd; 2018.

[15] Arakawa N, Bader LR (2022). Consensus development methods: considerations for national and global frameworks and policy development. Res Social Administrative Pharmacy: RSAP.

[16] McMillan SS, King M, Tully MP (2016). How to use the nominal group and Delphi techniques. Int J Clin Pharm.

[17] Shang Z (2023). Use of Delphi in health sciences research: a narrative review. Med (Baltim).

[18] Alberti S, Ferri P, Ghirotto L, Bonetti L, Rovesti S, Vannini V, Jackson M, Rossi F, Caleffi D (2023). The patient involvement in nursing education: a mixed-methods systematic review. Nurse Educ Today.

[19] Klemenc-Ketiš Z, Zafošnik U (2024). Interprofessional Education with simulations in Primary Care. Zdr Varst.

[20] Dong H, Lio J, Sherer R, Jiang I (2021). Some learning theories for medical educators. Med Sci Educ.

[21] Meredith C, Heslop P, Dodds C. Simulation: social work education in a third place. Social Work Educ 2021:1–18.

[22] Alrashidi N, Pasay an E, Alrashedi MS, Alqarni AS, Gonzales F, Bassuni EM, Pangket P, Estadilla L, Benjamin LS, Ahmed KE (2023). Effects of simulation in improving the self-confidence of student nurses in clinical practice: a systematic review. BMC Med Educ.

[23] Saleem M, Khan Z (2023). Healthcare Simulation: an effective way of learning in health care. Pak J Med Sci.

[24] Madsgaard A, Røykenes K, Smith-Strøm H, Kvernenes M (2022). The affective component of learning in simulation-based education - facilitators’ strategies to establish psychological safety and accommodate nursing students’ emotions. BMC Nurs.

[25] Alinier G, Oriot D (2022). Simulation-based education: deceiving learners with good intent. Adv Simul (London England).

[26] Nestel D, Gough S. Designing simulation-based learning activities: A systematic approach. In: *Healthcare Simulation Education* edn.; 2017: 135–142.

[27] Sawyer T, Eppich W, Brett-Fleegler M, Grant V, Cheng A (2016). More Than one way to debrief: a critical review of Healthcare Simulation Debriefing methods. Simul Healthcare: J Soc Simul Healthc.

[28] Klemenc-Ketis Z, Zafošnik U, Poplas Susič A (2020). An innovative approach to educating primary health care teams about medical emergencies. Educ Prim Care.

[29] Shepherd I, Burton T (2019). A conceptual framework for simulation in healthcare education - the need. Nurse Educ Today.

[30] Roche AF, Condron CM, Eppich WJ, O’Connor PE. A mixed methods study identifying the competencies of Health Care Simulation technicians. Simul Healthcare: J Soc Simul Healthc 2022. 10.1097/SIH.0000000000000682 PMC10581406 35940598

[31] Eppich W, Cheng A (2015). Competency-based simulation education: should competency standards apply for simulation educators?. BMJ Simul Technol Enhanced Learn.

[32] Ahmed RA, Cooper D, Mays CL, Weidman CM, Poore JA, Bona AM, Falvo LE, Moore MJ, Mitchell SA, Boyer TJ (2022). Development of a simulation technical competence curriculum for medical simulation fellows. Adv Simul (London England).

[33] Alhassan BA, Diebieri M, Anliengmene AA, Issah S (2023). A survey of knowledge and practice of simulation among health tutors in selected health training institutions. Nurs open.

[34] Brennan PA, Oeppen RS (2022). The role of human factors in improving patient safety. Trends Urol Men’s Health.

[35] Essex R, Weldon SM, Markowski M, Gurnett P, Slee R, Cleaver K, Stiell M, Jagodzinski L (2022). A systematic mapping literature review of Ethics in Healthcare Simulation and its methodological feasibility. Clin Simul Nurs.

[36] Park C, Murphy T. Healthcare simulationist code of ethics. In.: Society for simulation in healthcare; 2018.

[37] Buléon C, Mattatia L, Minehart RD, Rudolph JW, Lois FJ, Guillouet E, Philippon A-L, Brissaud O, Lefevre-Scelles A, Benhamou D (2022). Simulation-based summative assessment in healthcare: an overview of key principles for practice. Adv Simul.

[38] van der Vleuten CPM, Schuwirth LWT (2019). Assessment in the context of problem-based learning. Adv Health Sci Educ Theory Pract.

[39] Brazil V, Scott C, Matulich J, Shanahan B (2022). Developing a simulation safety policy for translational simulation programs in healthcare. Adv Simul (London England).

[40] Senvisky JM, McKenna RT, Okuda Y. Financing And Funding A Simulation Center. In: *StatPearls* edn. Treasure Island (FL) ineligible companies. Disclosure: Ryan McKenna declares no relevant financial relationships with ineligible companies. Disclosure: Yasuharu Okuda declares no relevant financial relationships with ineligible companies.: StatPearls Publishing LLC.; 2023. 33760545

[41] Jarelnape AA, Sagiron EI (2023). Evaluation of the effectiveness of Simulation-based teaching on nursing education: a systematic review. Egypt J Health Care.

[42] Lamé G, Dixon-Woods M (2020). Using clinical simulation to study how to improve quality and safety in healthcare. BMJ Simul Technol Enhanced Learn.

[43] Bajaj K, Brazil V, Purdy E (2023). Simulation as an improvement technique.

[44] Colman N, Doughty C, Arnold J, Stone K, Reid J, Dalpiaz A, Hebbar KB (2019). Simulation-based clinical systems testing for healthcare spaces: from intake through implementation. Adv Simul.

[45] Lei C, Palm K. Crisis Resource Management Training in Medical Simulation. edn. Treasure Island (FL) ineligible companies. Disclosure: Kenneth Palm declares no relevant financial relationships with ineligible companies. StatPearls Publishing LLC.; 2023. *StatPearls*. 31869172

[46] Parsons JR, Crichlow A, Ponnuru S, Shewokis PA, Goswami V, Griswold S (2018). Filling the gap: Simulation-based Crisis Resource Management Training for Emergency Medicine residents. Western J Emerg Med.

[47] Sollars ED, Xenakis N (2021). Simulation-based Continuing Education in Health Care Social Work: a Case Study of Clinical Training Innovation. Clin Soc Work J.

[48] Batt A, Williams B, Rich J, Tavares W (2021). A six-step model for developing competency frameworks in the Healthcare professions. Front Med.

