# Supplementary material for: Developing a competency framework for training with simulations in healthcare: a qualitative study
Source: BMC Med Educ. 2024 Feb 23;24:180. doi: 10.1186/s12909-024-05139-1 (PMC10893594; doi:10.1186/s12909-024-05139-1)
Supplement: Supplementary file 1 — The final model for basic and advanced level competencies and sub-competencies for the instructors providing basic and advanced levels of simulation-based training in healthcare [file 12909_2024_5139_MOESM1_ESM.docx]

Appendix 1: The final model for basic and advanced level competencies and sub-competencies for the instructors providing basic and advanced levels of simulation-based training in healthcare

| **Competence/Sub-competence** | **Advanced level** | **Basic level** |
| --- | --- | --- |
| 1. Knowledge of simulation training | X | X |
| 1. Is aware of the importance of simulation training | X | X |
| 1. Explains debriefing methods and tools | X | X |
| 1. Understands what fidelity means in simulation | X | X |
| 1. Explains high and low fidelity; augmented reality (VR), virtual reality (AR), physical fidelity; environmental fidelity; psychological fidelity | X | X |
| 1. Knows the guidelines for successful moulage | X | X |
| 1. Knows the guidelines for healthcare simulation training | X | X |
| 1. Knows the guidelines for in-situ simulation | X | X |
| 2. Education/Training Development | X | X |
| 1. Demonstrates how to develop scenarios for simulation education | X | X |
| 1. Develops a simulation training program | X | X |
| 1. Develops training for mobile simulation unit | X | X |
| 1. Develops training with simulated patient (SP) | X | X |
| 1. Develops training for in-situ simulation | X | X |
| 1. Develops health care simulation training guidelines | X | X |
| 3. Education/Training Performance | X | X |
| 1. Is able to prepare and give lectures | X | X |
| 1. Schedules and conducts debriefings | X | X |
| 1. Knows how to implement VR/AR standard for simulation education in healthcare | X | X |
| 1. Performs training in a mobile simulation unit | X | X |
| 1. Performs debriefing | X | X |
| 1. Performs in-situ simulation | X | X |
| 1. Performs high-fidelity simulation | X | X |
| 1. Performs training with SP | X | X |
| 1. Performs interprofessional simulation | X | X |
| 1. Performs simulation with emphasis on team collaboration | X | X |
| 4. Human Factors | X | X |
| 1. Considers human factors in preparing for simulation | X | X |
| 1. Is able to identify areas for improvement in the area of human factors | X | X |
| 1. Recognizes human factors in simulations | X | X |
| 1. Successfully prepares a simulation focusing on human factors | X | X |
| 1. Successfully conducts a simulation focusing on human factors |  | X |
| 5. Ethics in Simulation | X | X |
| 1. Understands the importance of ethical principles in the training process with simulations | X | X |
| 1. Identifies areas of need for ethical principles in the training process with simulations | X | X |
| 1. Conducts training with a high degree of psychological safety | X | X |
| 1. Conducts simulation according to ethical principles | X | X |
| 6. Assessment | X | X |
| 1. Understands the importance of evaluation principles | X | X |
| 1. Explains the Miller Pyramid for measuring the success of simulation training | X | X |
| 1. Explains the flowchart for developing a simulation-based evaluation | X | X |
| 1. Conducts a performance evaluation of training participants | X | X |
| 1. Analyse a performance evaluation of training participants |  | X |
| 7. Policies and Procedures | X |  |
| 1. Knows the example of policies and procedures in simulation training | X |  |
| 1. Explains the importance of good policies and procedures | X |  |
| 1. Creates policies and procedures for simulation training | X |  |
| 8. Organization and Coordination | X |  |
| 1. Understands the importance of and ways to reuse equipment | X |  |
| 1. Recognizes the benefits of working with a mobile simulation unit | X |  |
| 1. Understands the importance of embedding the simulation into a program | X |  |
| 1. Knows the model for embedding simulation into a program | X |  |
| 1. Creates the simulation centre’s annual plan | X |  |
| 1. Creates the simulation centre’s annual program that provides sufficient funding for costs, staff, and development | X |  |
| 1. Knows various accreditation standards | X |  |
| 1. Creates a plan for implementing accreditation of simulation centre | X |  |
| 1. Knows the terms related to space in simulation centre: human space; simulated environments; technical province | X |  |
| 1. Knows the activities that take place in the peripheral areas of the simulation centre | X |  |
| 1. Knows the importance of continuing medical education | X |  |
| 1. Knows the methodology of testing systems | X |  |
| 9. Research | X |  |
| 1. Understands the importance of research in simulation-based education | X |  |
| 1. Names the areas of research in health care simulations | X |  |
| 1. Plans a research methodology | X |  |
| 1. Conducts research | X |  |
| 1. Writes articles | X |  |
| 1. Knows the value of evidence-based medicine | X |  |
| 10. Quality Improvement | X |  |
| 1. Explains what quality improvement is | X |  |
| 1. Explains what safety of patients means | X |  |
| 1. Explains methods of quality improvement | X |  |
| 1. Understands the term quality indicators | X |  |
| 1. Recognizes most common areas for safety incidents in health care | X |  |
| 1. Is able to prepare a simulation based on real safety incidents’ data | X |  |
| 11. Crisis Management | X |  |
| 1. Understands the importance of preparing for a crisis and preventing a crisis | X |  |
| 1. Independently prepares a simulation on the topic of Crisis Management in Healthcare | X |  |
